# Gene expression vs. sequence divergence: comparative transcriptome sequencing among natural *Rhinolophus ferrumequinum* populations with different acoustic phenotypes

**DOI:** 10.1186/s12983-019-0336-7

**Published:** 2019-09-13

**Authors:** Hanbo Zhao, Hui Wang, Tong Liu, Sen Liu, Longru Jin, Xiaobin Huang, Wentao Dai, Keping Sun, Jiang Feng

**Affiliations:** 1 Jilin Provincial Key Laboratory of Animal Resource Conservation and Utilization, Northeast Normal University, Changchun, 130117 China; 2 Institute of Resources & Environment, Henan Polytechnic University, Jiaozuo, 454000 China; 3 Vector Laboratory, Institute of Pathogens and Vectors, Branch of Yunnan Provincial Key Laboratory for Zoonosis Control and Prevention, Dali University, Dali, 671003 China; 4 College of Life Science, Jilin Agricultural University, Changchun, 130118 China

**Keywords:** *Rhinolophus ferrumequinum*, Echolocation, Geographic evolution, Adaptation, Transcriptome

## Abstract

**Background:**

Although the sensory drive hypothesis can explain the geographic variation in echolocation frequencies of some bat species, the molecular mechanisms underlying this phenomenon are still unclear. The three lineages of greater horseshoe bat (*Rhinolophus ferrumequinum*) in China (northeast, central-east, and southwest) have significant geographic variation in resting frequencies (RF) of echolocation calls. Because their cochleae have an acoustic fovea that is highly sensitive to a narrow range of frequencies, we reported the transcriptomes of cochleae collected from three genetic lineages of *R. ferrumequinum*, which is an ideal organism for studying geographic variation in echolocation signals, and tried to understand the mechanisms behind this bat phenomenon by analyzing gene expression and sequence variation.

**Results:**

A total of 8190 differentially expressed genes (DEGs) were identified. We identified five modules from all DEGs that were significantly related to RF or forearm length (FL). DEGs in the RF-related modules were significantly enriched in the gene categories involved in neural activity, learning, and response to sound. DEGs in the FL-related modules were significantly enriched in the pathways related to muscle and actin functions. Using 21,945 single nucleotide polymorphisms, we identified 18 candidate unigenes associated with hearing, five of which were differentially expressed among the three populations. Additionally, the gene *ERBB4*, which regulates diverse cellular processes in the inner ear such as cell proliferation and differentiation, was in the largest module. We also found 49 unigenes that were under positive selection from 4105 one-to-one orthologous gene pairs between the three *R. ferrumequinum* lineages and three other Chiroptera species.

**Conclusions:**

The variability of gene expression and sequence divergence at the molecular level might provide evidence that can help elucidate the genetic basis of geographic variation in echolocation signals of greater horseshoe bats.

## Background

Many evolutionary biologists strive to explain the mechanisms of phenotypic divergence [1]. However, what drives phenotypic divergence remains one of the least understood biological phenomena [2]. Some evidence has suggested that different habitats are likely to impose different selective pressures on isolated populations and result in geographic variation of phenotypic traits, such as morphological, physiological, behavioral, and sensory traits [3, 4]. Elucidating the genetic mechanisms behind phenotypic divergence that are driven by adaptation is a primary mission of modern evolutionary biology [5].

Sensory traits in animals directly impact individual fitness by affecting resource acquisition, orientation, mate choice, and species recognition. Geographic variation in these traits is usually mediated by adaptive processes rather than random processes like genetic drift [6]. Therefore, the sensory drive hypothesis, which predicts a close association between the geographic variation of sensory signals and environmental variables, has been proposed to explain how environments affect signal traits and sensory systems [7].

Acoustic signals, which are an important sensory characteristic, have long attracted numerous researchers who mainly focused on the geographic variation of calls in birds, anurans, insects, and bats [8–10]. In particular, the echolocation calls of bats have drawn attention from an increasing number of researchers as a new research model for testing the sensory drive hypothesis. The high variability in echolocation calls over the distributional ranges of many lineages is likely due to numerous factors, including differences in environmental conditions, prey size, variation in body size, age, and sexual dimorphism [11–14]. Besides those external factors, internal molecular mechanisms also play an important role in the geographic variation of acoustic signals, because phenotypic changes indicate changes in gene expression, and genotypes can affect gene expression based on interactions between genotypes and the environment [15]. Although studies that evaluated intraspecific gene expression and genotype variation are scarce, some studies have found that several genes, such as *prestin* [16], *TMC1* [17], *KCNQ4* [18], *CDH23*, *PCHH15*, and *OTOF* [19], were related to the adaptation of echolocation and convergently evolved in some animals that use echolocation. However, the molecular mechanism underlying environmentally driven adaptive trait divergence of acoustic signals within species remains unclear.

The greater horseshoe bat (*Rhinolophus ferrumequinum*), a widespread, constant frequency–frequency modulating (CF–FM) bat, is an ideal model organism to help elucidate the molecular mechanisms underlying acoustic geographic variation and adaptive evolution of Chiroptera for several reasons. First, *R. ferrumequinum* has a broad distribution in the Old World and exhibits acoustic geographic variation. Different populations have different dominant frequencies of echolocation calls that have been reported [20, 21]. Second, Sun et al. [22] revealed a geographic pattern of *R. ferrumequinum* echolocation pulses emitted at rest (resting frequencies, RF), and, interestingly, they found that RF corresponded to genetic differentiation. Three genetic lineages, northeast (NE), central-east (CE), and southwest (SW), were identified based on several neutral genetic markers [22–24] and had significantly different echolocation calls. Third, combined with molecular data, acoustic parameters, and climatic factors, Sun et al. [22] inferred that ecological selection and cultural drift were most likely related to the variation of *R. ferrumequinum* calls across China. However, the internal molecular mechanisms of acoustic variation are still not known. For horseshoe bats, RF is largely genetically determined [25], so the divergence of echolocation calls between geographical populations might be related to genotype changes and differential gene expression.

Furthermore, the mammalian cochlea is an exceptionally sensitive organ that detects a very wide variety of sound intensities. The strongest intensity that does not damage the ear is 10^12^ larger than the threshold level of detectible sound, and can discriminate both infrasonic and ultrasonic sounds in different species [26]. Therefore, the cochlea is an important organ that participates in the hearing process by receiving acoustic signals. For bats, the specialization of cells in the ears and brain improves the response to the frequencies of the sounds they emit. *Rhinolophus ferrumequinum*, as a CF–FM bat, has cochleae with a specific area known as an acoustic fovea. The acoustic fovea is well-known for its key role in the process of Doppler-shift compensation behavior, because it makes the bats highly sensitive to a narrow range of frequencies [27, 28]. Although no study can definitively support that bat populations with different RF have different genetic features of the cochleae, significant genetic differences were detected between bat species with different RF in the cochleae [29]. Moreover, a study that focused on the cochleae of inbred mice with hearing variation revealed the existence of anatomical- and frequency-specific genes, and also demonstrated that there is at least one specific genome-wide locus that is significantly associated with each frequency in inbred mouse strains [30]. Even though there was no clear evidence that difference in RF of populations was mirrored by genetic differences in the cochlea, it would be appropriate to test the cochlea to reveal molecular mechanisms of acoustic variation of bats because of the role of the cochlea in hearing.

To explore the molecular mechanisms of the diversity of echolocation calls, we conducted RNA sequencing (RNA-Seq) in this study. Among novel sequencing technologies, RNA-Seq is outperforming the traditional hybridization-based microarray method, because it can evaluate gene expression without prior knowledge of the sequence [31, 32]. Furthermore, RNA-Seq can reveal genotype information and does not require prior genomic or genetic resources, and can be used as a cheaper alternative that enables identification of single nucleotide polymorphisms (SNPs) located in transcribed regions [33]. Recently, it has become easier to apply this approach to many organisms because of the development of appropriate statistically grounded pipelines for data analysis [34, 35]. Moreover, a recent study have demonstrated that small sample sizes are sufficient to assess interpopulation divergence when thousands of biallelic SNP markers were used [36]. Therefore, high-throughput genomic sequencing of RNA has great potential to elucidate evolution in non-model organisms.

In this study, we performed transcriptome sequencing of *R. ferrumequinum* cochlea samples collected from representative populations of three genetic lineages in China. We propose that the diversity of echolocation calls among *R. ferrumequinum* populations is, to a large extent, related to (i) regulation of gene expression and (ii) sequence divergence. Therefore, we analyzed gene expression and orthologous gene sequences to identify genes that may be involved in shaping the geographic variation of acoustic signals. Our results will provide insight into the underlying mechanism that produces geographic variation in echolocation calls.

## Methods

### Sample collection and phenotyping

We set mist nets to capture bats at dusk in unproductive habitats and collected 14 individuals in total from all three populations, which included representatives of all three clusters based on our previous study [22]. To reduce the impact of gender and age on gene expression, we sampled adult males of similar sizes and weights. We captured five individuals from a representative population of the NE genetic lineage in Jilin Province (sample IDs JL01–JL05, E 125.89°, N 43.28°) and one population of the CE lineage in Henan Province (sample IDs HN01–HN05, E 113.94°, N 35.71°). In Yunnan Province, we collected four individuals from a representative population of the SW lineage (sample IDs YN01–YN04, E 99.86°, N 26.53°). All sampling was conducted during the summer (late July to late August 2017).

The echolocation calls of greater horseshoe bats emitted at rest have simple structures and are ideal for sonographic analysis. Additionally, these calls have a narrow range of frequencies that are consistent with the maximal sensitivity of frequencies of the cochlear acoustic fovea. Therefore, we recorded calls emitted at rest using Avisoft-UltraSoundGate (Avisoft Bioacoustics, Glienicke, Germany) with a sample rate of 441 kHz at 16 bits/sample. We put the microphone (CM16/CMPA, Avisoft Bioacoustics, Berlin, Germany; flat frequency response: 10 Hz–200 kHz, ± 3 dB) approximately 30 cm in front of bats at rest, and the recordings were transferred to and saved on a computer.

We selected high-quality calls based on the criteria proposed by Russo et al. and Jiang et al. [37, 38] and analyzed them using Avisoft-SASLab Pro v 5.2.12 [39]. More specifically, initial calls were not considered for analysis, and only the second call per group was chosen when calls were emitted in groups. Moreover, we excluded calls with a CF portion that lasted less than 10 ms. For each bat, 30 high-quality calls were arbitrarily selected and measured for the CF components in the dominant second harmonic from the power spectra of a call. Then, the mean RF value of each individual was used in the analysis. Given that body size may act on acoustic features of bat echolocation calls, we also measured the forearm length (FL) of each bat. Then, we pairwise compared average RF and FL values among the three populations by Mann–Whitney U test. After we finished recording echolocation sounds and measuring FL, bats were sacrificed, and their whole cochleae were separately preserved in RNAlater (Tiangen Biotech, Beijing, China) within 25 min post-mortem for RNA-Seq data generation. All samples were subsequently stored at − 80 °C until RNA extraction.

### RNA-Seq library preparation and sequencing

We homogenized each cochlea sample and extracted total RNA using a TRIzol Kit (Promega, Madison, WI, USA) according to the manufacturer’s instructions. After checking the amount of RNA degradation by RNase-free agarose gel electrophoresis and capillary electrophoresis with a 2100 Bioanalyzer (Agilent Technologies, Santa Clara, CA, USA), we performed a poly (A)-capture to remove rRNA; then, mRNA was reverse-transcribed into first-strand cDNA using random primers. Next, we synthesized the second-strand cDNA using DNA polymerase I, RNase H, dNTP, and second-strand buffer. After purification using a QiaQuick PCR Extraction kit (Qiagen, Hilden, Germany), the cDNA fragments were end-repaired, poly(A)-tailed, and ligated to Illumina sequencing adapters. The ligation products were -elected by agarose gel electrophoresis and PCR amplification. Sequencing was carried out on an Illumina HiSeq X Ten (Illumina, San Diego, CA, USA) with 2 × 150-bp paired-end reads. All raw reads were deposited in the NCBI Short Read Archive (SRA) Database under SRA accession PRJNA515764.

### Transcript assembly, quantification, annotation, and differential expression testing

First, we filtered and trimmed barcoded RNA-Seq reads and low-quality reads. We removed reads that were contaminated by Illumina adapters, contained more than 10% unknown nucleotides (N), or had more than 40% low-quality (Q-value ≤10) bases using trimmomatic v 0.36 [40]. Next, filtered reads from all 14 individual cDNA libraries, which included individuals of all the three groups, were loaded into Trinity v 2.4.0 to assemble a de novo reference transcriptome with default parameters. To avoid redundant transcripts, we kept the longest isoform for each “trinity gene” identified by Trinity using a Perl script and defined the longest transcript as a unigene. This de novo reference was used to obtain expression profiles. Moreover, we also assembled a reference transcriptome for each population in the same way for positive selection analysis. To evaluate and compare the completeness of the gene set of our four transcriptomes, we used Benchmarking Universal Single-Copy Orthologs (BUSCO v 3.1.0) to search for orthologs in the “laurasiatherian_odb9” database, which includes a collection of 6253 single-copy Laurasiatherian orthologs. All generated unigenes in these four unigene sets were aligned with an E-value of 1E− 5 to the following protein databases: Nr [41], Swiss-Prot [42], KEGG [43], and COG/KOG [42] by BLASTx [44].

We then performed pairwise comparisons to identify differentially expressed genes (DEGs) in the Bioconductor package edgeR with default parameters [45], which scored well in a recent comparison [46]. In this study, unigenes with false discovery rate (FDR) ≤ 0.05 and an absolute value of a log_2_-fold change > 1 were considered DEGs. More specifically, the de novo reference transcriptome assembled with all of the samples was used as the reference sequence, and we calculated the reads per kilobase per million mapped reads (RPKM) of each sample by mapping high-quality clean reads to the reference transcriptome using the short read alignment tool in Bowtie2 with default parameters [47]. According to the plot showing the sample relationships based on multidimensional scaling (MDS), we filtered outlier samples. Then, we performed pairwise comparisons for the three populations to select DEGs. For each comparison (HN vs. JL, HN vs. YN, and YN vs. JL), the former population always had a higher call frequency than the latter. We considered a unigene with higher expression in the former population to be upregulated and vice versa to be downregulated. The *P*-value was corrected by the Benjamini and Hochberg method [48]. We visualized the gene expression profiling of all DEGs by heatmap analyses of hierarchical clustering.

### Weighted gene co-expression network analysis

A weighted gene co-expression network analysis (WGCNA) [49] was used to identify unigenes and gene networks associated with RF and FL. First, we extracted the expression values of DEGs obtained from three pairwise comparisons and calculated Pearson’s correlation matrix for all genes. Next, the correlation matrix was transformed by raising all values to a soft-thresholding power (β = 9 in this study) to obtain an adjacency matrix. Then, we transformed the adjacency matrix into a topological overlap matrix (TOM) and identified modules using 1-TOM as the distance measure with a deepSplit value of 2 and a minimum size cut-off of 50 for the resulting dendrogram. After identification by clustering, groups of co-regulated genes (modules) were merged with a height cut-off of 0.25. Then, we searched biologically meaningful modules by evaluating Pearson’s correlation between modules and phenotypic features. We focused on those modules that were strongly correlated with the phenotypic features. Subsequently, to get a better understanding of how those genes impact phenotypic features, genes in all modules were studied using heatmap analyses of hierarchical clustering, GO functional enrichment, and KEGG pathway analysis (*P* < 0.01, FDR < 0.01). We also merged those modules that were significantly correlated with RF or FL to perform GO term enrichment analysis (*P* < 0.01, FDR < 0.01).

### SNP identification and filtering

As described in detail in Maestre et al. [34], SNPs were called from RNA-Seq data without a reference genome using KisSplice v 2.4.0 [35] and KisSplice2RefTranscriptome v 1.3.2 [34]. To remove sequencing errors, we filtered rare variants by setting the cut-off to 5%. Then, we aligned SNPs to the de novo reference transcriptome assembled with all of the samples using BLAT suite.34 [50] with default parameters to predict amino acid changes. The output file was manually converted to a VCF file, and the following filters were applied: 1) SNPs that were located in the noncoding region of transcripts were removed; 2) rare variants were filtered out, as they are more likely to be sequencing error, and only markers with MAF > 5% were retained; 3) only those SNPs with less than 10% missing data across all sites were included in further analyses; and 4) to reduce linkage, SNPs were pruned using the “snpgdsLDpruning” function in the R package SNPRelate v 1.18.1 with a linkage disequilibrium threshold of 0.1. The filtered VCF file was converted into the formats supported by software used in further analyses with PGDSpider v 2.1.1.5 [51].

### Environmental variables

Climate variable layers were obtained from the CHELSA dataset [52], which includes 35 years of high spatial and temporal accuracy bioclimatic data (from 1979 to 2013). Given the effect of temperature and relative humidity on echolocation call frequency [22, 53], 19 climate variables related to temperature and precipitation (BIO01–BIO19, Additional file 1: Table S1) were extracted by the R package ‘raster’ [54] for each sampling site. First, we performed an association analysis between climate variables. Because of the high correlation between many of the climate variables, a principal component analysis (PCA) was performed to summarize the environmental data and identify the data that explained the highest proportion of the environmental variance. The first principal component (PC) for climate variables was used as the climate variables in gene–environment association (GEA) analyses.

### Discovery of SNPs putatively under divergent selection

Detected SNPs under selection are likely to have high false-positive rates caused by inaccurate population structure control or spatial autocorrelation of allele frequencies. Recently, use of a combined analysis approach has been increasing to obtain more robust results [55–58]. We used two different genome scan methods to detect loci putatively under divergent selection: outlier tests and GEA analyses. An outlier test was implemented in the program PCAdapt v 3.1.0 [59]. After choosing the number of PCs, the test statistic was computed based on the number K of PCs (in our case, K = 2). Based on the correlations between SNPs and the first two PCs, we computed and corrected *P*-values by Bonferroni correction (α < 0.1) [60].

The programs Bayenv2 and LFMM v 1.5 were used to perform the GEA test. For Bayenv2 [61], 100,000 MCMC cycles were first run to obtain a covariance matrix. Bayenv2 used five independent runs with 50,000 iterations to estimate Bayes factors (BF) for each SNP, and the SNPs with an average BF > 3 were considered to be associated with the first climate variable PC. In LFMM [62], the number of latent factors that best described the population structure in the dataset was set based on the results of STRUCTURE v 2.3.4 [63, 64], Tracy–Widom tests were implemented in SmartPCA in EIGENSOFT v 6.1.4 [65] using the mean genomic inflation factor (λ) [66]. LFMM was run five times, and, as suggested in Frichot & François 2015 [67], we re-adjusted the *P*-values using the expected value of the FDR equal to q = 10%. Finally, we applied functional enrichment analyses for the outliers predicted using OmicShare tools [68] to test for over-representation of GO and KEGG functional categories.

### Identification of one-to-one orthologous genes

Genomic protein sequences of *R. sinicus*, *Hipposideros armiger*, and *Pteropus alecto* were downloaded from NCBI. We obtained the longest transcript of each gene using a Python script and identified one-to-one orthologous genes among three *R. ferrumequinum* populations and three other specie*s* using the best reciprocal hit method (E-value, 1E− 5). For each predicted single-copy orthologous gene, we performed multiple alignments with PRANK v 170,427 (parameters: -f = FASTA -F -codon -noxml -notree -post) [69]. To reduce the rate of false-positive predictions, Gblocks v 0.91b [70] was used to filter out sequencing errors, incorrect alignments, and non-orthologous regions based on codons with the following parameters: -t = C -b3 = 1 -b4 = 6 -B5 = N. After trimming, only the alignments with lengths greater than 100 bp were used for further analysis.

### Phylogenetic tree and positive selection analyses

The phylogenetic tree was constructed using concatenated sequences of all filtered single-copy orthologous genes common to the three populations of greater horseshoe bat and three other bat species. Maximum likelihood analyses were run using the JTT + I + G + F model in RAxML v 8.2.10 [71], and relative support of internal nodes was assessed by rapid bootstrap (−f a –× 12,345) of 1000 replicates.

Using our tree topology as the guide tree, branch-site model in PAML4 (parameters: null hypothesis: model = 2, NSsites = 2, fix_omega = 1, omega = 1; alternative hypothesis: model = 2, NSsites = 2, fix_omega = 0, omega = 1) [72] was used to detect positive selection in the one-to-one orthologous genes. The three *R. ferrumequinum* populations were used as the foreground branches. We used a likelihood ratio test to detect positive selection on each foreground branch, and the genes with FDR less than 0.05 were considered positively selected genes (PSGs). After identifying PSGs, the empirical Bayes method was implemented to calculate posterior probabilities and record positively selected sites. We also performed enrichment analysis on the PSG dataset.

## Results

### Transcriptome assembly, quantification, and annotation

After transcriptome quality filtering, we obtained approximately 4 Gb of clean data for each of the 14 cDNA samples from the Illumina platform. For each sample, we obtained 26–36 million paired-end reads. After quality filtering, approximately 96% of the raw reads remained, and the sequencing result details are provided in Additional file 1: Table S2. We used Trinity methods with standard parameter values for the de novo assemblies of all samples and the three separate populations. Assembly of all samples yielded 70,704 transcripts that ranged from 201 to 27,462 bp with an N50 of 2532 bp. For the assembly of the three separate populations, we obtained 53,558–103,932 transcripts with N50 s from 2250 to 2811. Annotation results of unigenes in the four reference transcriptomes are provided in Additional file 1: Table S3. The completeness of the four transcriptomes was assessed using the BUSCO pipeline, which revealed that the majority of the Laurasiatherian core genes had been successfully recovered in all four assemblies. Of the 6253 single-copy Laurasiatherian orthologs, 78.0–84.0% were complete, 6.7–10.3% were fragmented, and 9.3–11.7% were missing (Table 1). The good coverage indicated that all four assemblies were high-quality. For all four unigene sets, almost half had annotation hits to *R. sinicus* (Additional file 1: Table S4).

**Table 1:** Transcriptome completeness inferred from Benchmarking Universal Single-Copy Orthologs (BUSCO) search. ALL, JL, YN, and HN represent de novo references used to obtain expression profiles, single nucleotide polymorphisms (SNPs), and the reference transcriptome for each separate population. The number and percentage represent the number of genes inferred from BUSCO and their percentage of all 6253 single-copy Laurasiatherian orthologs

BUSCO statistic	ALL	JL	YN	HN
Complete BUSCOs	5252 (84.0%)	4880 (78.0%)	5067 (81.0%)	5159 (82.5%)
Complete - single-copy BUSCOs	5170 (82.7%)	4734 (75.7%)	4996 (79.9%)	5027 (80.4%)
Complete – duplicated BUSCOs	82 (1.3%)	146 (2.3%)	71 (1.1%)	132 (2.1%)
Fragmented BUSCOs	416 (6.7%)	647 (10.3%)	489 (7.8%)	427 (6.8%)
Missing BUSCOs	585 (9.3%)	726 (11.7%)	697 (11.2%)	667 (10.7%)

### Phenotypic variation and differential gene expression between populations

The significant differences in RF were detected among the three *R. ferrumequinum* populations (Mann–Whitney U test, *P* < 0.05 in all cases). The HN population had the highest RF (mean = 75.5 kHz, SD = 0.36), followed by the YN population (mean = 73.0 kHz, SD = 0.93); the JL population (mean = 68.2 kHz, SD = 0.32) had the lowest RF (Additional file 2: Figure S1 a). For FL, the YN population had the longest FL (mean = 61.22 mm, SD = 1.90), followed by the HN population (mean = 60.08 mm, SD = 0.55) and then the JL population (mean = 58.60 mm, SD = 0.58). A significant difference was detected between HN and JL (Mann–Whitney U test, *P* = 0.008) (Additional file 2: Figure S1 b).

**Fig. 1:**
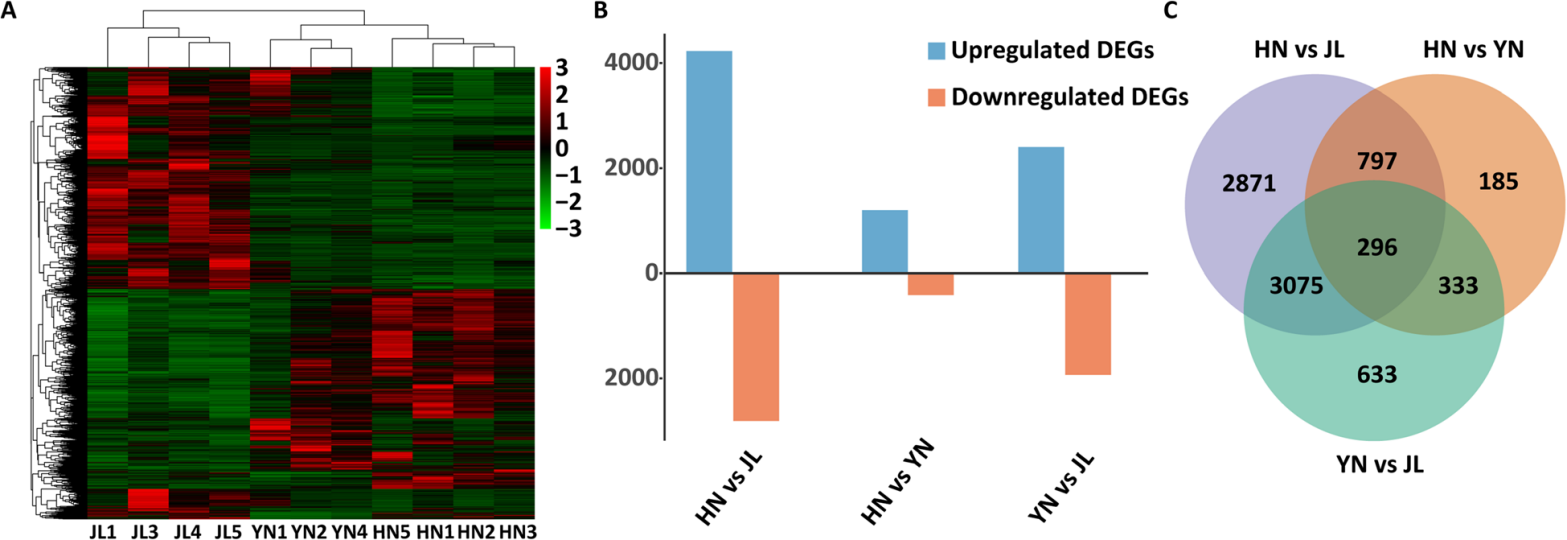
Differential gene expression analysis of three populations. **a** Heatmap depicting 8190 differentially expressed genes (DEGs). Upregulated and downregulated genes are indicated in red and green, respectively; their expression patterns clustered, and their transcription levels are depicted as logFC values. **b** Numbers of upregulated and downregulated genes based on pairwise comparison. **c** Venn diagram showing the number of DEGs between each two comparisons and the number of shared DEGs

The pairwise comparisons showed significant gene expression differences among the three populations. Three samples, JL3, HN4, and YN2, were removed as outliers based on the MDS plot (Additional file 3: Figure S2). Based on the remnant samples, 8190 total DEGs were obtained from pairwise differential expression analysis, and the heatmap of the hierarchical clustering of all DEGs indicated that HN was more similar to YN than to JL based on gene expression patterns (Fig. 1a). For each comparison, a total of 7039, 4337, and 1611 DEGs in HN vs. JL, YN vs. JL, and HN vs. YN were detected, respectively (Fig. 1b, c).

### Correlation between gene network modules and phenotype

**Fig. 2:**
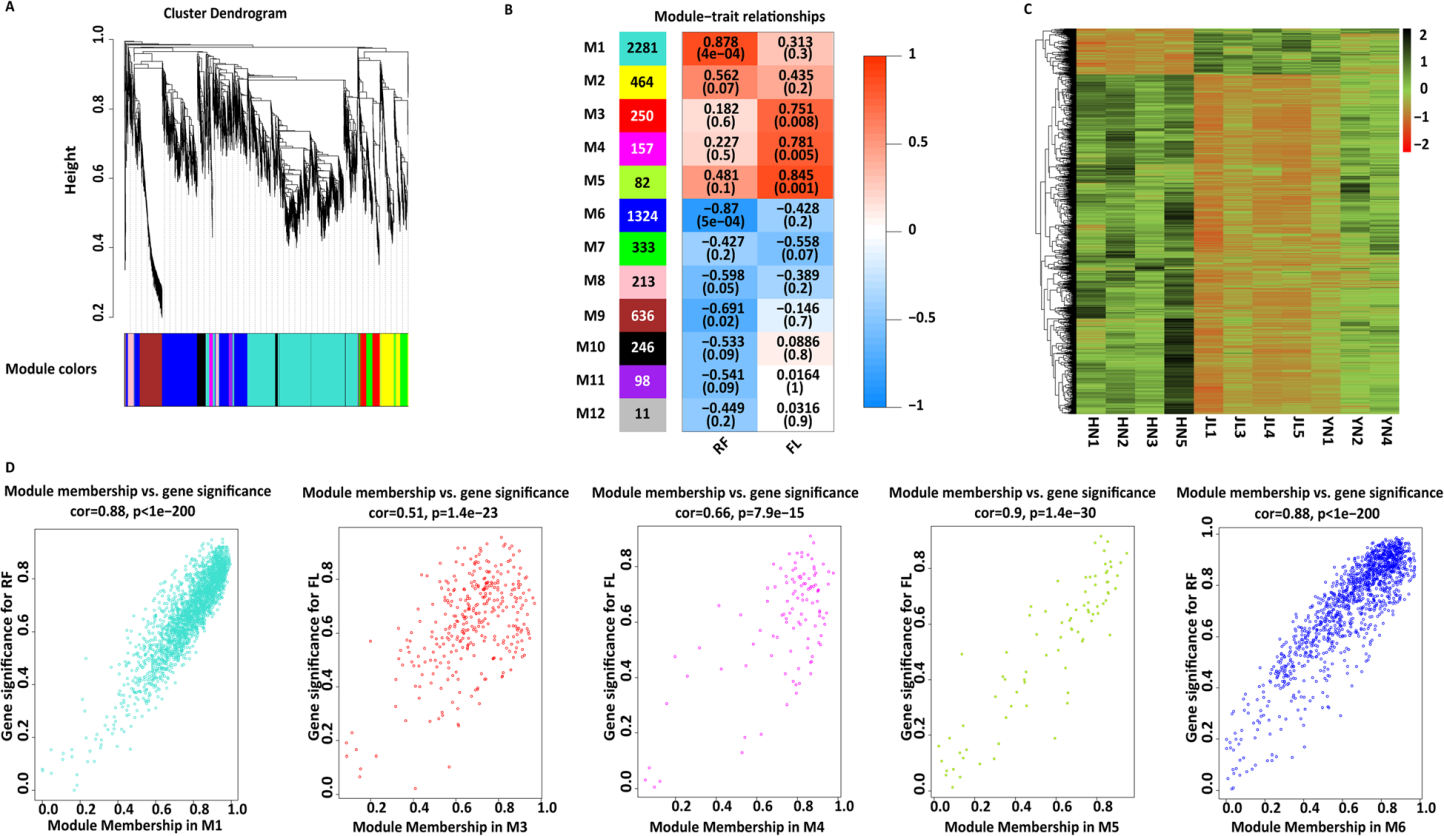
WGCNA applied to 8190 differentially expressed genes. **a** Hierarchical clustering of co-expression data. **b** Table of module–trait relationships. Resting frequency and forearm length are shown on the X-axis. The value at the top of each square represents the correlation coefficient between the module eigengene and the trait with the correlation *P*-value in parentheses. The left panel shows 12 modules and the number of their genes. The right panel is a color scale for module trait correlation from − 1 to 1. **c** Heatmap summary and hierarchical clustering of genes in M1. The hierarchical clustering was generated using Spearman’s correlation coefficients of log_2_-transformed reads per kilobase per million mapped reads of expression values. Rows are standardized; red indicates high values and green indicates low values. **d** Scatterplot of the intramodular analysis (module membership versus gene significance) of genes found in M1, M3, M4, M5, and M6

Based on all 8190 DEGs (Fig. 1c), a gene co-expression network was constructed, and 12 gene modules were created by WGCNA (Fig. 2a). Two of them (M1, M6) were most significantly correlated with RF (both *P* < 0.001), and three modules (M3, M4, and M5) were significantly correlated with FL (all *P* < 0.01, Fig. 2b). M1, with 2281 unigenes, had the highest number of DEGs, and the heatmap of hierarchical clustering indicated large differences in gene expression between the three populations (Fig. 2c). We plotted scaled connectivity on the X-axis and gene significance (absolute value of the correlation coefficient, r, between gene expression and RF or FL) on the Y-axis for each module to visualize the relationships and significant positive correlations between gene significance and intramodular connectivity (Fig. 2d).

**Fig. 3:**
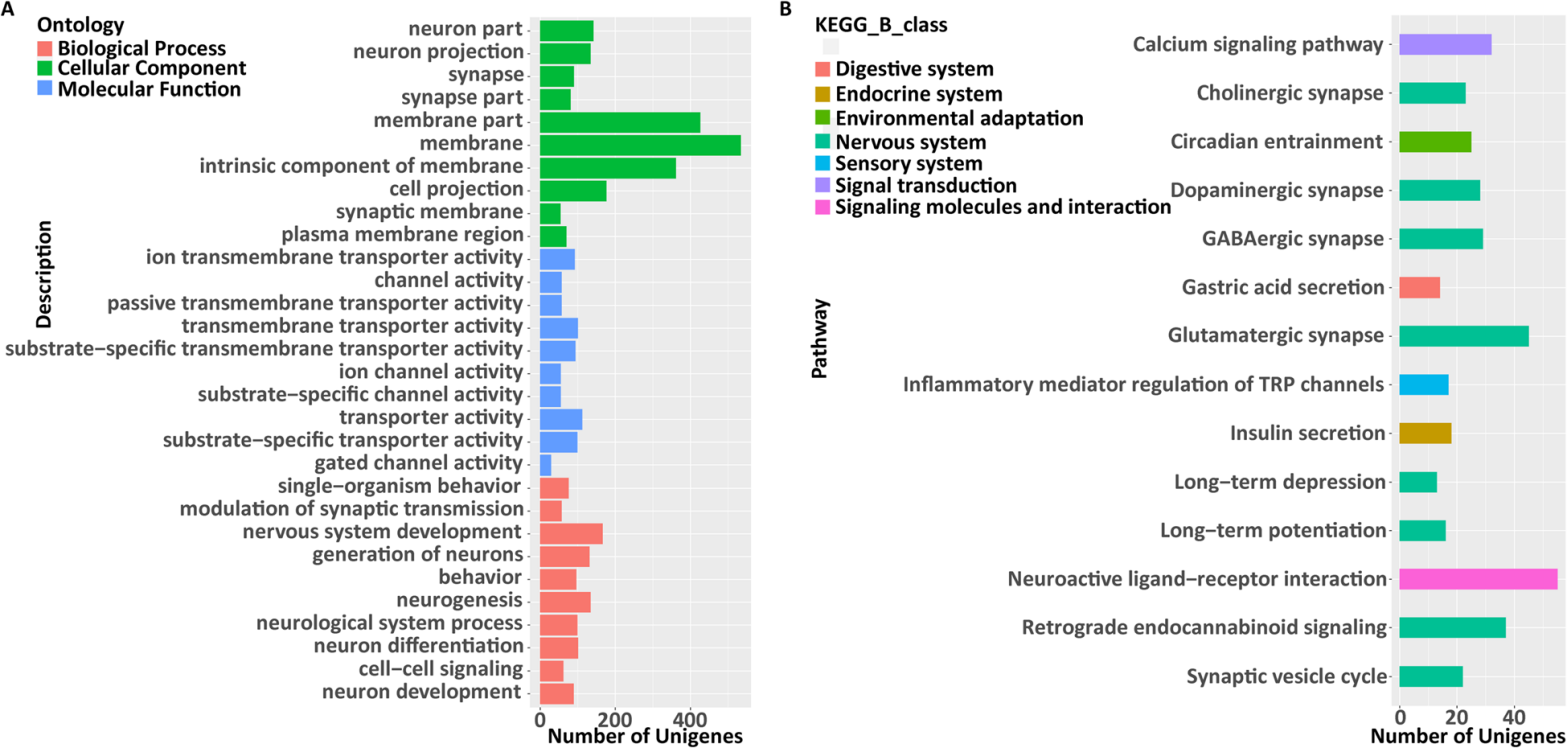
GO and KEGG enrichment analyses of genes in M1. **a** Top 30 enriched GO terms. **b** All enriched KEGG pathways

To obtain a clearer understanding of how differential gene expression patterns affect acoustic characteristics and body size, we then performed GO term and KEGG pathway enrichment analyses for each gene module. For unigenes included in M3, M4, M5, and M6, GO and KEGG enrichment analysis did not reveal any significantly enriched gene profile directly related to hearing (Additional file 1: Table S5 and Table S6). Unigenes in the M1 were significantly enriched in GO classifications that covered all three domains of ontology and were related to synaptic, neuron, membrane, and ion transporter functions, such as “synapse” (GO:0045202, FDR = 1.17E− 35), “neuron part” (GO:0097458, FDR = 1.25E− 43), “membrane part” (GO:0044425, FDR = 1.11E− 33), and “ion channel complex” (GO:0034702, FDR = 1E− 15) (Fig. 3a and Additional file 1: Table S5), and the KEGG pathway was also significantly enriched in synaptic functions (Fig. 3b and Additional file 1: Table S6). We also found that GO items related to learning were significantly enriched, such as “learning or memory” (GO:0007611, FDR = 1.01E− 17) and “learning” (GO:0007612, FDR = 2.83E− 07).

For unigenes in RF-related modules (M1 and M6), the GO term enrichment analysis results were similar, but two of those GO terms were associated with the response to sound: “response to auditory stimulus” (GO:0010996, FDR = 6.10E− 3) and “auditory behavior” (GO:0031223, FDR = 6.10E− 3) (Additional file 1: Table S7). For unigenes in FL-related modules (M3, M4 and M5), statistically significant items were related to a variety of functions, and many items were related to muscle or actin, such as “muscle system process” (GO:0003012, FDR = 6.30E− 14), “striated muscle cell development” (GO:0055002, FDR = 8.91E− 12), and “actin filament-based movement” (GO:0030048, FDR = 8.01E− 7) (Additional file 1: Table S8).

### Adaptive loci

**Fig. 4:**
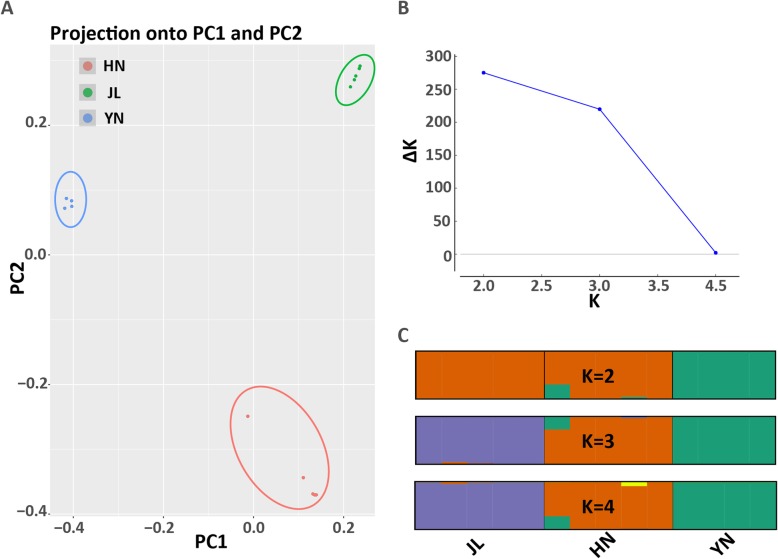
Genetic differentiation among the three greater horseshoe bats populations. **a** PCA plot of PC1 vs. PC2 from 21,945 SNPs for all 14 samples. Populations are colored according to genetic group assignment. **b** Ad-hoc statistics Δ(K) based on STRUCTURE lnP(D) summarized over 10 replications for each K (assumed number of populations). **c** Population structure for K = 2–4. Vertical lines indicate separate clusters, with cluster colors indicating various ancestries

After applying the filtering criteria as described in the Methods, we identified 21,945 SNPs distributed across 17,772 unigenes for use in further analyses. PCA of the 21,945 SNPs revealed two dimensions that clustered by population (JL, HN, and YN; Fig. 4a). The Δ(K) from the STRUCTURE run showed that lnP(D) was highest when K = 2 (Fig. 4b), but the bar-plot for K = 2–4 showed that K = 3 represented the best possible number of populations (Fig. 4c). In LFMM, we chose K = 2 based on the Tracy–Widom results, because K = 2 had λ estimates closer to 1.0 than K = 3.

**Fig. 5:**
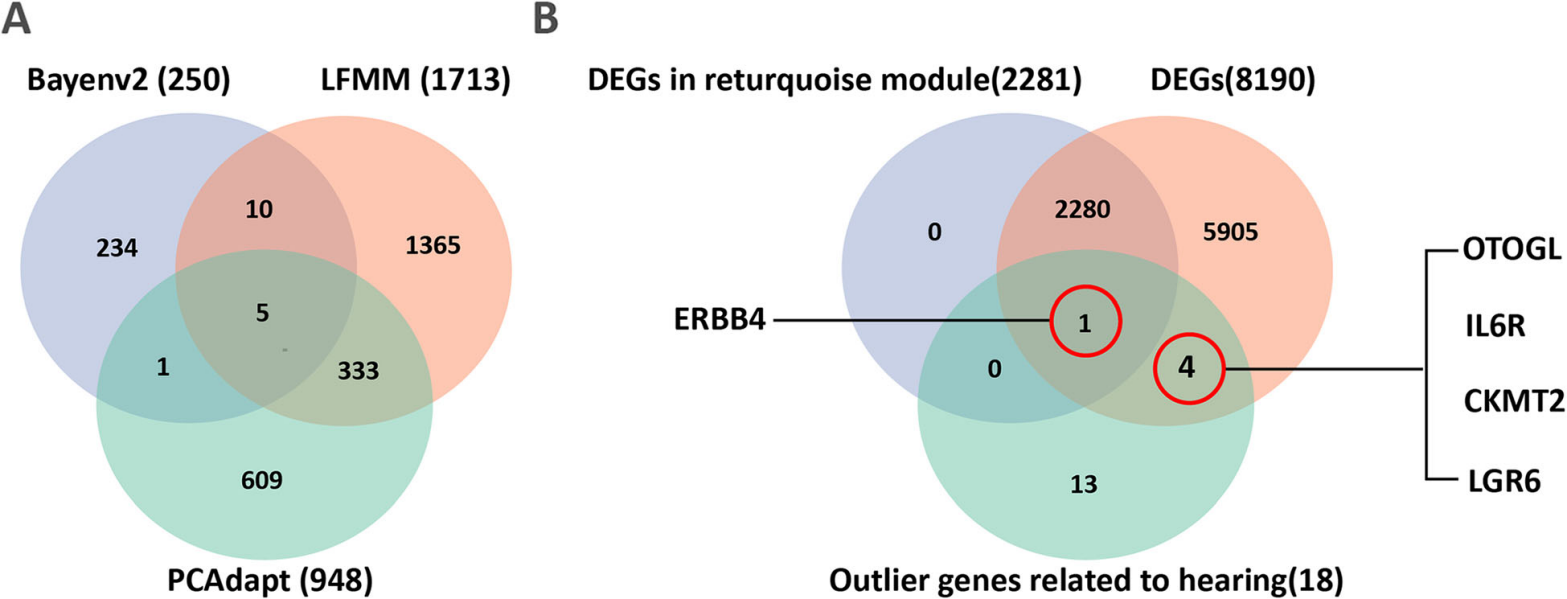
Venn diagrams illustrating the overlap in different outlier detection methods and DEGs. **a** Venn diagram of loci putatively detected as being under adaptive divergence. **b** Venn diagram of outlier genes and DEGs

To explain most of the climate variation, PCA was performed to reduce the variables into fewer components. In our case, climate variations were highly consistent, and the contribution of the first PC was over 85% (Additional file 4: Figure S3). The association analysis of SNP markers and the first PC identified 250 and 948 unique SNP markers using Bayenv2 and LFMM, respectively. We also identified an extensive list of 1713 outliers using PCAdapt. Only 349 significant SNP markers were identified by at least two of the three outlier tests (Fig. 5a), and this finding indicated that some of these SNP markers were significantly associated with environmental variation. We predicted that these SNPs fell within 349 unigenes, and 310 of these significant SNP markers had annotations in the greater horseshoe bat transcriptomes (Additional file 1: Table S9). The identified unigenes represented a broad range of biological processes, such as immune (i.e., immunoglobulin superfamily member 3-like), transcription (i.e., aquaporin-3), and synaptic (i.e., synaptotagmin-16-like) processes. Furthermore, 18 of these unigenes were annotated in 18 genes that were thought to be involved in hearing-related processes or development of the hearing organ. Five of these auditory genes, *ERBB4* (Unigene0004747), *OTOGL* (Unigene0036784), *IL6R* (Unigene0051178), *CKMT2* (Unigene0004892), and *LGR6* (Unigene0005403), were significantly differentially expressed among the three populations, and *ERBB4* (Unigene0004747) was in the M1 of WGCNA (Fig. 5b).

### Orthologous gene prediction and constructed phylogenetic tree

**Fig. 6:**
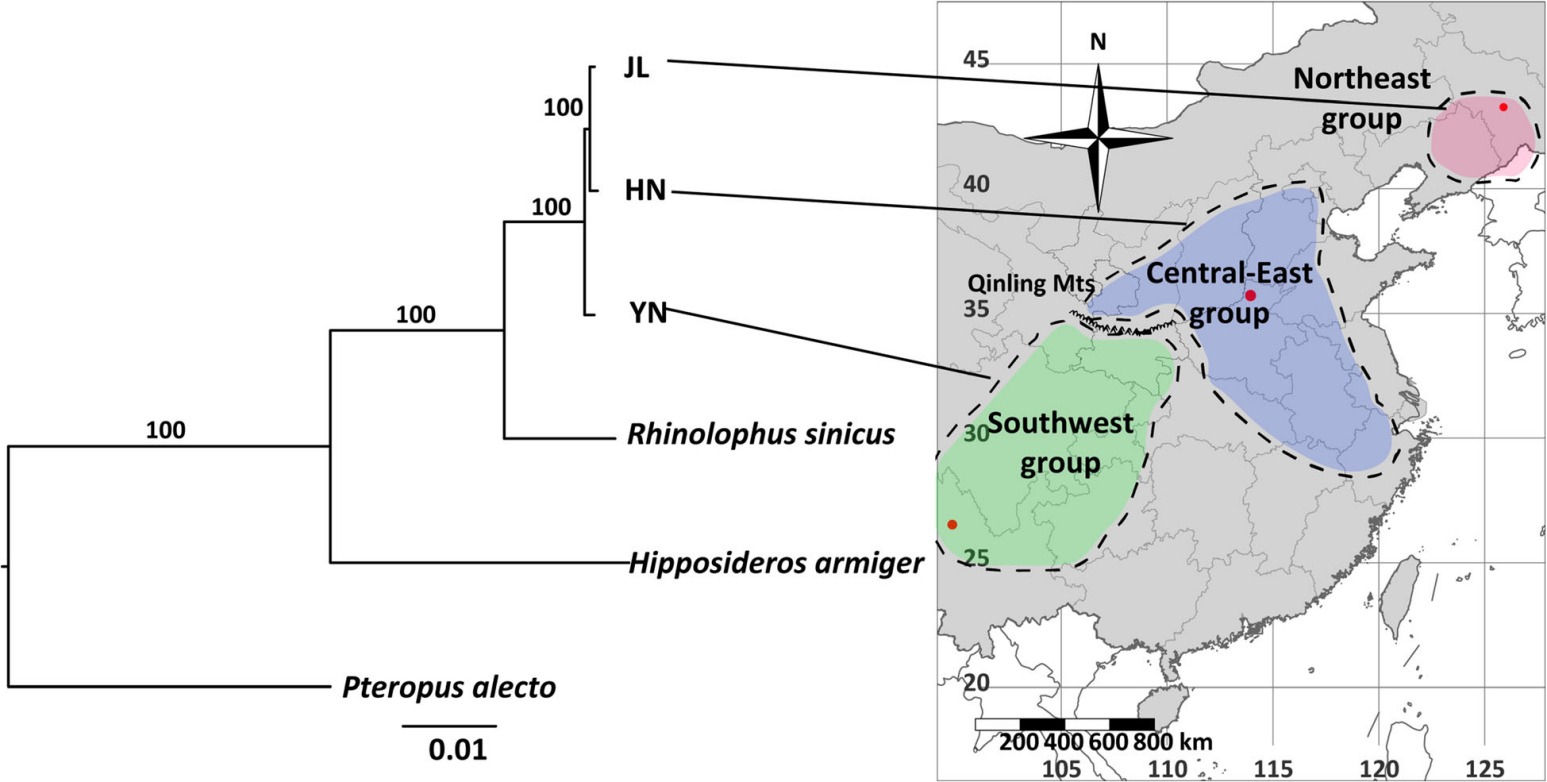
Phylogenetic relationships of four bat species based on all single-copy orthologous genes. A map of China was superimposed over to show the biogeographic ranges for each greater horseshoe bat population (from Sun et al. [22]), and the sampling sites are shown by red dots. The percentage of bootstrap replicates that supported each node is shown above the branch

After removing low-quality sequences, we identified 4151 one-to-one orthologous gene pairs in the three populations, black fly fox (*P. alecto*), great roundleaf bat (*H. armiger*), and Chinese horseshoe bat (*R. sinicus*). After multiple sequence alignment and filtering, we retained 4105 one-to-one orthologous genes for model selection. All of these genes were concatenated into a single supergene dataset for model selection. The generated maximum likelihood tree of the three greater horseshoe bat lineages and other bat species was determined to be well resolved based on the high bootstrap value (100%). The phylogenetic relationship of those species indicated that JL was more closely related to HN than YN, and this was consistent with the findings of previous research [22] (Fig. 6).

### Positive selection

We detected PSGs among the 4105 orthologous genes along the JL, HN, and YN branches using our reconstructed tree topology, and detected 31, 6, and 12 PSGs, respectively (Additional file 1: Table S10). The PSGs had a variety of functions, such as in cilia formation, oxidoreductase activity, and immune process. The enrichment analyses of candidate PSGs showed that no category was significantly enriched.

## Discussion

Recently, geographic variation in echolocation frequencies of bats has been frequently observed and widely investigated in the context of allopatric differentiation [4]. According to the sensory drive hypothesis, acoustic signals vary in association with local climatic conditions, and animal communication systems are adapted to local environments. Different environmental conditions may cause phenotypic and gene expression diversity between different populations, such as humans (*Homo sapiens*) [73] and fruit flies (*Drosophila melanogaster*) [74]. However, we know little about the molecular mechanisms underlying geographic variation of acoustic signals, especially in non-model organisms. Here, we report high-quality cochlea transcriptomic data for three representative populations of three Chinese *R. ferrumequinum* lineages*.* The quality of our sequencing allowed us obtain precise information about gene expression, sequence variation, and orthologous genes (Table 1). Our study will help elucidate genes or sequences with differential expression among populations that might be closely related to bat RF and could provide insight into the genetic basis of geographic variation in acoustic signals.

### DEGs related to acoustic signal traits

In this study, we found that gene expression diversity among different populations and gene expression of cochleae could contribute to the RF variation of *R. ferrumequinum* echolocation calls. First, we found a significant change in gene expression patterns among the three populations (Fig. 1a), and we obtained a large number of DEGs using pairwise comparison methods (Fig. 1b and c). Second, HN and YN populations had the lowest difference in RF (difference of average RF = 2.5 kHz) and more similar gene expression patterns than the other two pairwise comparisons (HN vs JL and YN vs JL; Fig. 1a), even though this finding was not consistent with the phylogenetic relationship inferred from orthologous genes (Fig. 6) and the STRUCTURE results based on SNPs (Fig. 4b). Third, we used WGCNA, a very widely used R software package, to identify groups, known as modules, of correlated genes in suitable data [75]. We found strong associations between phenotypic traits (RF and FL) and five different expression gene modules (Fig. 2b). The unigenes with higher connectivity tended to be more strongly correlated with RF and FL. This finding indicated that these unigenes might play potentially important roles in the acoustic signal phenotype of bats.

Although we could not confirm which unigenes affect RF variation, our enrichment results showed that unigenes in M1 were enriched in pathways related to nervous system activity, such as a neuron, synapse, membrane, transporter, and channel activity, which are crucial in nerve transmission in processes such as hearing. Several studies have shown that the same neural circuit, “the song system,” controls the song of all songbirds, and genes activate the singing pattern [76–78]. Therefore, our results could indicate that the variable echolocation calls among different populations are regulated by the expression of genes related to the nervous system that adapt to different environments. We also identified potentially new GO categories for geographic variation in echolocation frequencies (learning or memory, and learning), as they were significantly enriched in our differentially expressed RF-related gene modules. Although there is no direct evidence that the greater horseshoe bat has song-learning abilities, Sun et al. suggested the possibility of cultural drift of the greater horseshoe bat [22]. Therefore, we cannot rule out the possibility that these bats have song-learning abilities. Although unigenes in the other four modules were significantly enriched in several GO items or KEGG pathways, their functions were not directly related to hearing, potentially because environmental factors and the state of the individuals could influence the expression of those unigenes. However, the expression changes of those unigenes might also affect hearing in some unknown ways. Therefore, we should not exclude the possibility that those unigenes could affect hearing before performing additional function analyses.

Interestingly, the enrichment results of unigenes in RF-related modules (M1 and M6) indicated another possibility: that the expression of genes related to sound response could affect bat RF. In channel catfish (*Ictalurus punctatus)*, which can hear at higher frequencies, the genes in the pathway related to sound response were highly expressed in auditory organs, and could be associated with hearing [79]. Although experimental studies on the genes of these GOs are still limited, it would be interesting to study how the differential expression of those genes affect hearing. For unigenes in FL-related modules, many of the significantly enriched pathways were related to muscle and actin. Though we have no direct proof that FL is related to muscle mass, the regulation of skeletal muscle cell growth and proliferation can cause variation in body size [80], which could influence bat RF.

### Outlier loci and genes under positive selection

We also attempted to identify the genetic basis of geographic variation in echolocation frequencies of bats based on nucleotide variations. In general, larger sample sizes are thought to be better for population genetic studies [81]. However, another studied showed that, even with a very small sample size (i.e., two individuals), accurate estimates of Fst could be obtained with a large number of SNPs (≥ 1500) [82]. For threatened species, species with reduced population sizes, and species that are difficult to obtain, such as bats, it is hard and detrimental to their populations to obtain enough samples. Therefore, high-throughput screening technologies are promising for estimating genetic diversity and differentiation in such populations from very small sample sizes or populations undergoing reduction.

In this study, we found 349 candidate loci identified by at least two outlier tests. The outliers from Bayenv2, LFMM, and PCAdapt included 1.14, 4.31, and 7.81% of the total number of loci analyzed from our RNA-Seq dataset, respectively; these percentages are similar to the candidate coverage reported in other studies (2.52, 4.87, and 7.52%, respectively) using at least one of the same software programs [83, 84]. The overall percentage of outliers may be affected by species and the dataset. More specifically, the factors that determine the dataset, such as false-positive rate, sampling, genome size, power, and composition, would influence the numbers of SNPs and then affect the coverage of outliers [85]. The three methods may yield different results because they theoretically differ. Therefore, those genes recognized by more than one method are more likely to be under selection. Although there were not many candidate loci detected by overlap of the three outlier tests, especially between Bayenv2 vs. LFMM, Bayenv2 vs. PCAdapt, and all three methods, those 349 candidate loci detected by more than one method are likely important in adaptation.

Of all 349 candidate loci, 18 were in the unigenes annotated in 18 genes that are related to maintaining normal cochlea or neurofunction. Of the 18 genes, *PSAP* (Unigene0000367) [86], *ERBB4* (Unigene0004747) [87, 88], *LGR6* (Unigene0005403) [89], *BAK1* (Unigene0040895) [90], *OSTF1* (Unigene0005552) [91], and *TSHZ1* (Unigene0008250) [92] are necessary for hearing, with functions involved in the maintenance of nerve cell functions, hair cell protection, and skeleton formation. Mutations, deletions, or gene expression changes of *MUC19* (Unigene0008431) [93], *IL-6* (Unigene0051178) [94], *ZNF469* (Unigene0005986) [95], *CKMT2* (Unigene0004892) [96], *TMC2* (Unigene0035276) [97], *PYCR2* (Unigene0032863) [98], and *OTOGL* (Unigene0036784) [99] could lead to hearing loss. *ATE1* (Unigene0043244) [100], *TPRN* (Unigene0030527) [101], *TRIOBP* (Unigene0033824) [102], *GREB1* (Unigene0021168) [103], and *DPY19L2* (Unigene0017291) [104] are thought to be related to nonsyndromic hearing impairment by damaging structures in the inner ear. Moreover, five of these unigenes were differentially expressed, and Unigene0004747 (annotated *ERBB4*) was in the turquoise module and significantly related to RF (Fig. 5b). Although the mechanisms underlying how the environment affects those genes among bat populations remain unclear, functions of the genes indicated their importance in hearing, especially those differentially expressed mutant genes. In the echolocation process, those genes might affect sensitivity to calls of specific frequencies, their echolocation signals, by regulating neural activity or inner ear structure, which receives these signals. Moreover, it cannot be ruled out that the geographic variation of acoustic signals is directly or indirectly influenced by other candidate loci.

The three populations might have undergone differential selection in different environments, because genes under positive selection varied in each lineage (Additional file 1: Table S10). For the genes under positive selection, we did not identify any enriched GO term or pathway. Moreover, we do not have direct evidence that those PSGs had functions related to hearing, but we cannot rule out the possibility that the candidate genes directly or indirectly influence greater horseshoe bat hearing.

### Limitations of this study

Our results from several analytical methods demonstrated that there are distinct DEGs and sequence divergences among populations that exhibit geographic variation of acoustic signals, and the expression or sequence divergences of these genes may be related to the geographic variation in echolocation frequencies. However, as is typical for many non-model studies, our results were constrained by several technical and statistical factors. For bats, we had to collect samples from the wild where neither ecological conditions nor age can be strictly controlled. Moreover, the particularity of bats limits both sampling design and sample size, which can affect the identification of candidate genes, and many methods are known to be susceptible to false positives. By taking these factors into account, we were unable to definitively determine which candidate genes directly or indirectly affect bat hearing and RF without further verification. Although we can only speculate until further physiological studies are conducted, our evidence indicates that these genes and gene groups may be important in shaping the geographic variation of signal structure, and those genes will be the focus of a future study.

## Conclusions

Using representative populations of the greater horseshoe bat in China, we obtained transcriptome data of *R. ferrumequinum* cochleae and analyzed gene expression and sequence data to examine gene expression changes and genotypes that contribute to geographic variation in echolocation calls. We identified some DEG modules and genomic variation among different populations related to RF or environment variables that could influence calling. The DEGs in modules that were significantly related to RF or FL were significantly associated with neurological and muscular functions, such as synaptic function, neuronal function, ion channel function, response to sound, learning behavior, muscle, and actin functions. As researchers pointed out in a previous study [22], both environmental adaptation and song learning are related to acoustic characteristics of greater horseshoe bats. Additionally, muscle mass, which could be related to body size, might also affect RF. Although specific genes could not be pinpointed, our results still indicated the potential importance of those genes. Genotype variation provided another way to understand the geographic variation of acoustic signals, and outlier loci and genes under positive selection might also be related to features of echolocation calls. Some of those genes have been reported to be related to hearing; five of them were significantly differentially expressed among populations, and *ERBB4* was in the module that was significantly correlated with RF. This finding indicated the importance of these genes, although the function of particular amino acid substitutions in these genes is unknown. In this study, even if RF could partially explain the changes in gene expression and sequence variation, future functional experiments will be necessary to validate their importance. Although there were limitations of our sampling strategy and analyses, our results partially explained the intraspecific geographic variation of acoustic signals and provided a direction for future research.

## Supplementary information

**Additional file 1: Table S1.** Summary of investigated climate and environmental variables. **Table S2.** Summary of Trinity de novo transcriptome assembly statistics of *Rhinolophus ferrumequinum.* **Table S3.** Annotation result statistics between unigenes and databases. **Table S4.** Nr functional annotation of four unigene datasets hits to *Rhinolophus sinicus* and other species. **Table S5.** Complete results of the Gene Ontology (GO) enrichment analysis for gene sets in modules defined by Weighted gene co-expression network analysis (WGCNA). **Table S6.** Complete results of the Kyoto Encyclopedia of Genes and Genomes (KEGG) enrichment analysis for gene sets in modules defined by WGCNA. **Table S7.** Complete results of the GO enrichment analysis for the merged gene set in modules significant associated with RF. **Table S8.** Complete results of the GO enrichment analysis for the merged gene set in modules significantly associated with FL. **Table S9.** Outlier loci (*N* = 349) identified in two tests for selection. ‘SNP position’ shows the position in the contig that contains the outlier loci. ‘Tests’ shows which tests identified the SNP as an outlier. **Table S10.** Detail of genes under positive selection.

**Additional file 2: Figure S1.** Boxplot of RF (a) and FL (b) between three populations. For each box plot, the box represents the 0.25 quantile, median, and 0.75 quantile. On either side of the box, the whiskers extend to the minimum and maximum values. *, indicates a statistically significant difference between populations. ° and ◇ represent outliers and means, respectively.

**Additional file 3: Figure S2.** Multidimensional scaling plot of 14 samples generated with edgeR. The axes represent gene expression levels among the three different populations. The samples in the red circles were removed from gene expression analysis.

**Additional file 4: Figure S3.** Scree plot of the principal component analysis. The first dimension explained 87.2% of the variance.

## Data Availability

The datasets used and/or analyzed during the current study are available from the corresponding author on reasonable request. All data generated or analyzed during this study are included in this published article and its supplementary information files.

## Abbreviations

BUSCO: Benchmarking Universal Single-Copy Orthologs
CE: Central-east
CF–FM: Constant frequency–frequency modulating
DEGs: Differentially expressed genes
FL: Forearm length
GEA: Gene-environment association
MDS: Multidimensional scaling
NE: Northeast
PC: Principal Component
PCA: Principal component analysis
PSGs: Positively selected genes
RF: Resting frequency
RNA-Seq: RNA sequencing
RPKM: Reads per kilobase per million mapped reads
SNPs: Single nucleotide polymorphisms
SW: Southwest
TOM: Topological overlap matrix
WGCNA: Weighted gene co-expression network analysis
λ: mean genomic inflation factor
