# Supplementary figures and images for: Gene expression vs. sequence divergence: comparative transcriptome sequencing among natural Rhinolophus ferrumequinum populations with different acoustic phenotypes
Source: Front Zool. 2019 Sep 13;16:37. doi: 10.1186/s12983-019-0336-7 (PMC6743130; doi:10.1186/s12983-019-0336-7)

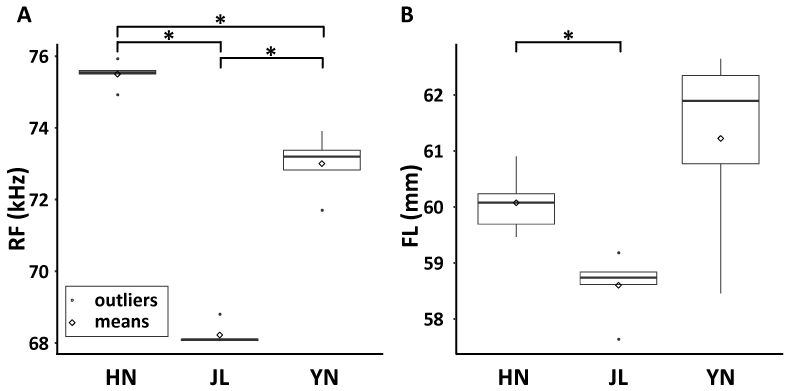

Supplement: Supplementary file 2 — Additional file 2: Figure S1. Boxplot of RF (a) and FL (b) between three populations. For each box plot, the box represents the 0.25 quantile, median, and 0.75 quantile. On either side of the box, the whiskers extend to the minimum and maximum values. *, indicates a statistically significant difference between populations. ° and ◇ represent outliers and means, respectively. [file 12983_2019_336_MOESM2_ESM.tif]

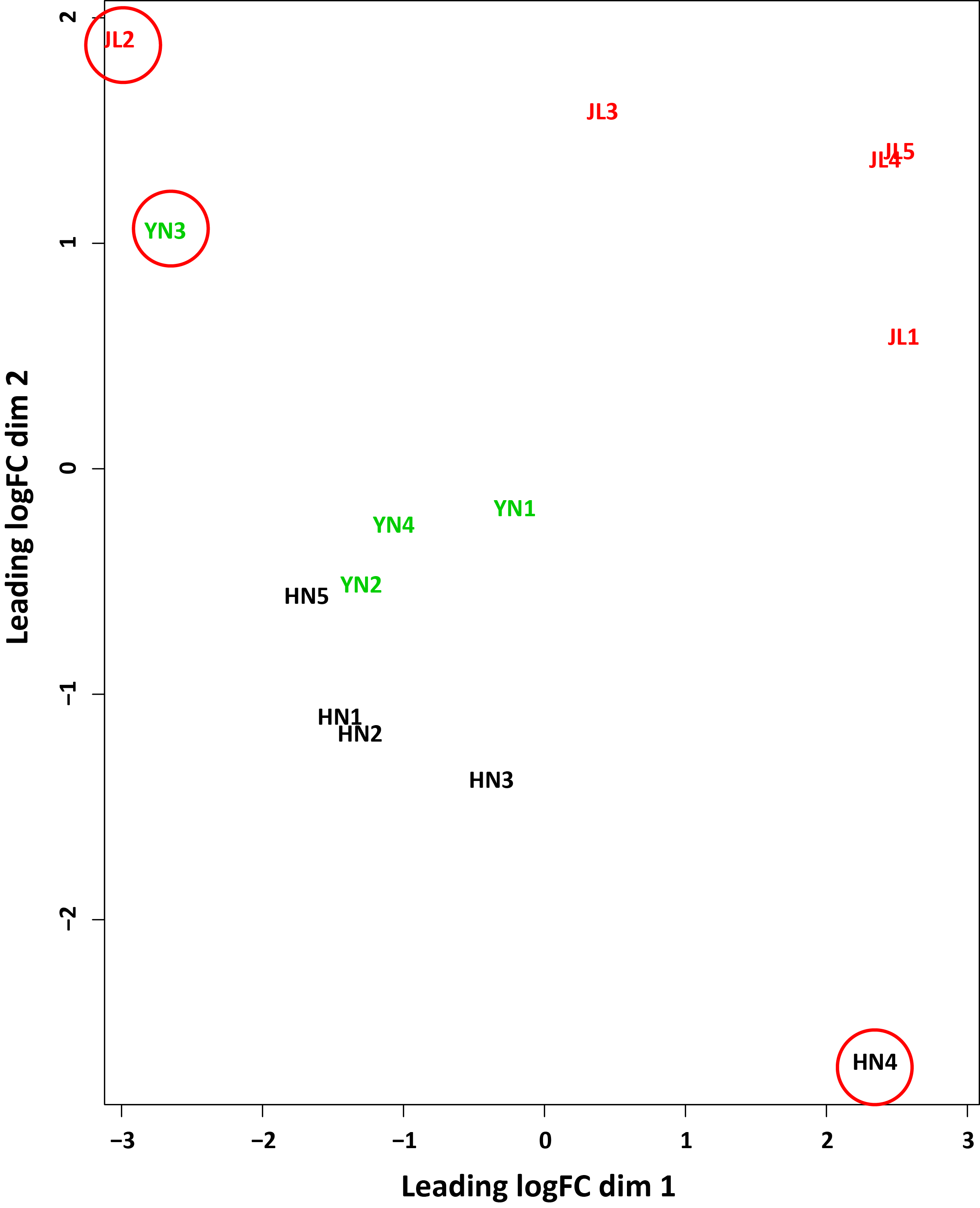

Supplement: Supplementary file 3 — Additional file 3: Figure S2. Multidimensional scaling plot of 14 samples generated with edgeR. The axes represent gene expression levels among the three different populations. The samples in the red circles were removed from gene expression analysis. [file 12983_2019_336_MOESM3_ESM.tif]

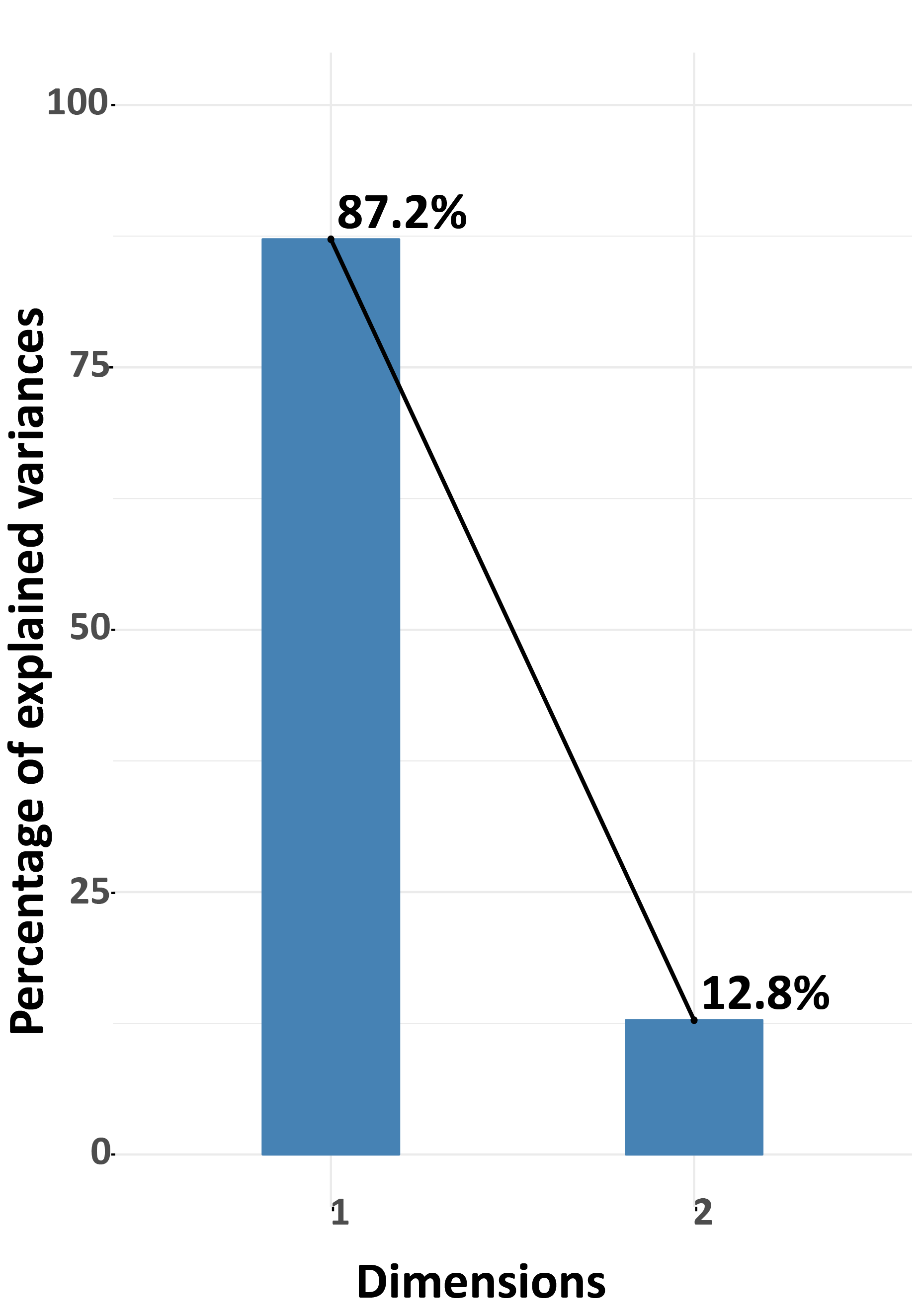

Supplement: Supplementary file 4 — Additional file 4: Figure S3. Scree plot of the principal component analysis. The first dimension explained 87.2% of the variance. [file 12983_2019_336_MOESM4_ESM.tif]
